# Acceptability of 100-mg moxifloxacin in children with rifampicin-resistant TB in three high-burden countries

**DOI:** 10.5588/ijtldopen.25.0365

**Published:** 2025-10-10

**Authors:** N. Suryavanshi, H.R. Draper, G. Dhumal, S. Bagchi, N.T. Castillo-Carandang, A. Marthinus, A.M.A. Cheong, A. Kinikar, M. Paradkar, A. Gupta, J.D.R. Ocampo, M.V.G. Frias, D.J.O. Casalme, A. Hesseling, A.J. Garcia-Prats, M. Palmer, G. Hoddinott, L. Viljoen, on behalf of the CATALYST Trial Team

**Affiliations:** ^1^ Byramjee Jeejeebhoy Government Medical College, Johns Hopkins Clinical Research Site, Pune, India;; ^2^ Desmond Tutu TB Centre, Department of Paediatrics and Child Health, Stellenbosch University, Stellenbosch, South Africa;; ^3^ Department of Clinical Epidemiology, College of Medicine, University of the Philippines, Manila, Philippines;; ^4^ De La Salle Health Sciences Institute, Dasmariñas City, Philippines;; ^5^ Department of Paediatrics, Byramjee Jeejeebhoy Government Medical College, Pune, India;; ^6^ Department of Infectious Disease, School of Medicine, Johns Hopkins University, Baltimaore, MD, USA;; ^7^ Department of Pediatrics, School of Medicine and Public Health, University of Wisconsin, Madison, WI, USA;; ^8^ School of Public Health, Faculty of Medicine and Health, The University of Sydney, Sydney, NSW, Australia.

**Keywords:** tuberculosis, palatability, dispersible drug, drug-resistant, DR-TB

## Abstract

**BACKGROUND:**

Routinely, a 400-mg tablet of moxifloxacin is used in children with rifampicin-resistant TB (RR-TB), but it has very poor acceptability. We describe the acceptability of a 100-mg dispersible moxifloxacin among children and their caregivers in South Africa, India, and the Philippines.

**METHODS:**

This study is nested in a pharmacokinetics, safety, and acceptability trial of new formulations of clofazimine and moxifloxacin in children with RR-TB. Quantitative and qualitative data were collected at four time points over 24 weeks and were analysed descriptively and thematically.

**FINDINGS:**

Median age of participants (n = 36) was 4.9 years. Children and caregivers from all three countries preferred the dispersible 100-mg moxifloxacin to the routine 400-mg tablet due to the relative ease of administration. The 100-mg formulation was unpalatably bitter. Children who were able to swallow the 100-mg formulation preferred to do so. The smaller size of the 100-mg tablets enhanced their ease of preparation and acceptability, although some older participants experienced the increase in the number of tablets (compared with single 400-mg tablet) as a burden.

**CONCLUSION:**

The 100-mg moxifloxacin dispersible formulation is preferred over 400-mg. Overall, moxifloxacin palatability remains sub-optimal, and there is a need to further improve the acceptability of RR-TB treatments for children.

In 2022, an estimated 1.25 million children developed TB,^1^ with nearly half being <5 years. Among the 25,000–32,000 children having multidrug-resistant or rifampicin-resistant TB (RR-TB), only 12%–16% were diagnosed and treated.^2–4^ RR-TB treatment remains challenging and is associated with long-term adverse effects.^5,6^ There have, however, been many recent advancements with shorter, injectable-free regimens utilising new and repurposed TB drugs now available and recommended by World Health Organization (WHO).^5,7^ There is increasing awareness that acceptability of medications may affect children’s and caregivers’ treatment experience, adherence, and thus treatment success.^8,9^ Key component of medication acceptability in children includes acceptance of the taste, smell, size/volume, and texture of a medication.^8,10^ Moxifloxacin is a high-priority drug recommended by the WHO for use in both short and longer regimens for the treatment of RR-TB.^6^ Until recently, oral moxifloxacin was only available as a 400-mg tablet, which is challenging to administer to younger children who require lower doses necessitating splitting and/or crushing of the tablet^11^ and is a difficult compound to taste-mask because of its bitterness. A few studies reported that majority of children disliked the taste-masked suspension of moxifloxacin.^12,13^

The analysis presented here involved eliciting the experience of children, and their caregivers, with a specific focus on the acceptability of a 100-mg moxifloxacin (Macleods Pharmaceuticals, Mumbai, India), nested in the CATALYST study.^14^

## METHODS

CATALYST was a phase I/II trial of the pharmacokinetics, safety, and acceptability of new formulations of clofazimine and moxifloxacin in children treated for RR-TB conducted between March 2021 and July 2023 in South Africa, India, and the Philippines.^14^ Data were collected from children treated for RR-TB in Cape Town, South Africa; Pune, India; and the provinces of Cavite and Laguna in the Philippines.

### Population

Children between 0 and <15 years old, confirmed or clinically diagnosed with RR-TB through TB programmes, living with or without HIV, treated with both clofazimine and a fluoroquinolone as part of a RR-TB treatment, and on treatment for <16 weeks were eligible to participate.

### Study design

A parallel mixed-method acceptability evaluation was undertaken. Children with RR-TB received weight-banded doses of moxifloxacin consistent with current WHO-recommended doses using 400-mg tablet and 100-mg dispersible tablet formulations. The 400-mg tablets were manufactured by either Macleods Pharmaceuticals, India, or Hetero Pharmaceuticals, India (excipients: lactose monohydrate, povidone K29/32, lactose [anhydrous], croscarmellose sodium, silica colloidal [anhydrous], and magnesium stearate; film coating: hypromellose, titanium dioxide [E171], macrogol 400, and iron oxide Red [E172]), depending on what was routinely available locally. The 100-mg dispersible moxifloxacin formulation was manufactured by Micro Labs, India (excipients: microcrystalline cellulose, croscarmellose sodium, magnesium stearate, colloidal anhydrous silica, pineapple flavour sucralose). After enrolment into the study, participants received the routinely available formulation of moxifloxacin (400-mg tablet) that had to be dissolved in water in order to administer the appropriate dose to young children. Participants were then switched to the study moxifloxacin formulation (100-mg dispersible tablet). This allowed for comparison of acceptability experiences between formulations. On the day of the first pharmacokinetic sampling visit (week 0), the 400-mg moxifloxacin was administered by crushing/splitting the whole tablet, mixing it with 10 mL of water, and giving the appropriate dose in millilitres. Outside of the pharmacokinetic sampling days, children and their caregivers administered the 400-mg moxifloxacin tablet at home using their preferred method (e.g., swallowing whole rather than mixing in water if preferred). After children switched to the 100-mg moxifloxacin tablet, this was administered by dispersing the correct number of tablets in 5 mL of water on pharmacokinetic sampling days and as per the child and caregiver’s preference on other days. Quantitative acceptability assessments were completed after a moxifloxacin dose was administered at a pharmacokinetic sampling visit (week 0) and at weeks 2, 8, and 24. A sub-sample of children–caregivers who agreed and provided informed consent was recruited in qualitative interviews. Participants could opt out of interviews without compromising trial participation.

### Data collection

Quantitative data were collected through standard questionnaires (Supplementary Data S1) administered by the trained study staff using electronic devices (tablets). Data were managed in REDCap software.^15^ The acceptability questionnaire was available in English, and it was translated in relevant local languages (Afrikaans or Xhosa in South Africa, Marathi or Hindi in India, and Tagalog in the Philippines) and was pilot tested to ensure fidelity among participants. The data collectors were multi-lingual and used structured and consistent questioning approaches. The qualitative data were collected through in-depth interviews (∼45 min each) with a purposively selected sub-sample of child-caregiver dyads over four interactions within the child’s 24-week treatment of 100-mg moxifloxacin using discussion guides (Supplementary Data S2) by female, post-graduate behavioural scientists having research experience of >5 years (NS, LV, NTC-C, AM, AMAC, JDRO, GD, and SB), and the interviews were audio-recorded. None of the child-caregiver dyads refused participation.

### Data analysis

Summary statistics were used to describe administration experiences related of 400- and 100-mg moxifloxacin. The quantitative acceptability data were described using frequencies, and percentages at weeks 0, 2, 8, and 24 and were dichotomised as strongly agree/agree/neutral versus disagree/strongly disagree. The data were compared using the McNemar test and analysed using Stata Special Edition 16.1 software.^16^

Qualitative data analysis followed a deductive thematic approach informed by the usability domain of Wademan et al.’s^17^ acceptability framework that includes dimensions of administration processes, palatability, and appeal. Cross-country case descriptions were coded and combined into a matrix by co-authors and cross-checked during a workshop led by the last author. Data are reported using the COREQ checklist (Supplementary Data S3).

### Ethical statement

The study was approved by the Health Research Ethics Committee at Stellenbosch University, South African Health Products Regulatory Authority (SAHPRA), BJGMC Ethics Committee in India, and De La Salle University, Research Ethics Office (DLSU-REO), in the Philippines. All caregivers (≥18 years old) provided written informed consent in their local language, and children between 8 and 14 years provided written assent.

## RESULTS

Acceptability questionnaires were collected from 36 children/caregivers. At study entry, child participants had median ages of 4.9 years (interquartile range: 0.4–15). Table 1 displays the demographics of the participants completing the questionnaires and qualitative interviews. The qualitative interviews were conducted with a subset of 26 child–caregiver dyads.

**Table 1.** Baseline demographics of study population: participants completing questionnaires and qualitative interviews.

Participants completing questionnaires	Total (n = 36)	South Africa (n = 20)	India (n = 6)	Philippines (n = 10)
Sex				
Male (%)	14 (38.9)	9 (45.0)	1 (16.7)	4 (40.0)
Female (%)	22 (61.1)	11 (55.0)	5 (83.3)	6 (60.0)
Age				
0–7 years old	27 (75.0)	19 (95.0)	3 (50.0)	5 (50.0)
8–14 years old	9 (25.0)	1 (5.0)	3 (50.0)	5 (50.0)

Participants completing qualitative interviews	Total (n = 26)	South Africa (n = 13)	India (n = 6)	Philippines (n = 7)
Sex				
Male	11	7	1	3
Female	15	6	5	4
Age				
0–7 years old	15	12	3	4
8–14 years old	11	1	3	3

### Usability domain of Wademan’s acceptability framework

#### Administration process: ease of administration

Participants in qualitative interviews reported that the 400-mg moxifloxacin was difficult to administer as it needs to be crushed and then dissolved in water which takes time, and consequently administration of the full dose of medicine was not always possible. The 100-mg moxifloxacin tablets could be dispersed in 5 mL of water and administered with a syringe, which was experienced as easier and convenient.

Those pills [400-mg moxifloxacin] that you needed to crush were difficult to administer. These [100-mg] ones dissolve quickly, so at least he can drink them now.

(SACTA08) Caregiver from South Africa

Various factors influence palatability, including taste, smell, and size.^18,19^

#### Taste and taste masking

Table 2 displays the frequencies and percentages of ingestion facilitation agents participants used with 400- and 100-mg moxifloxacin. In total, 83% (24/29) of participants reported grinding the 400-mg moxifloxacin and mixing it with liquid or food to facilitate administration while 78% (28/36) reported dispersing the 100-mg moxifloxacin in water to facilitate administration. Also swallowing 100-mg tablet whole is reported by 19.4% (7/36) and 400-mg tablet by 17 % (5/29).

My child prefers the new formulation of moxifloxacin [100-mg] [rated 4/5 compared to 1/5] even if it is bitter. She takes four tablets of the medicine at a time and swallows them whole to taste them less.

(PHCTC06) Caregiver from the Philippines

**Table 2.** 400-mg moxifloxacin administration at week 0 and 100-mg moxifloxacin administration at week 2.

	Routine moxifloxacin (week 0) (n = 29)	100-mg moxifloxacin (week 2) (n = 36)
How the 400-mg moxifloxacin is usually administered		
Swallowed whole (%)	5 (17.2)	
Chewed (%)	0	
Grind finely and mix with liquid (%)	22 (75.9)	
Grind finely and mix with food (%)	2 (6.9)	
How the 100-mg moxifloxacin formulation is usually administered		
Swallowed whole (%)		7 (19.4)
Chewed (%)		0
Dispersed in water (%)		28 (77.8)
Other (%)		1 (2.8)**^A^**

A Most often swallowed whole, but in one instance, the participant dispersed moxifloxacin in water.

Table 3 displays experiences of mixing items with 400- and 100-mg moxifloxacin to either facilitate administration or to improve taste. In total, 93% (27/29) and 64% (23/36) used water to facilitate administration of the 400- and 100-mg moxifloxacin, respectively. Sweetening agents such as yoghurt, honey, and sugar were mixed with the 400-mg moxifloxacin by 10.3% (3/29) of participants and with 100-mg moxifloxacin by 22% (8/36) participants.

I do not like to take that tablet [100-mg moxifloxacin] with banana. Once I tried it with yoghurt. I take tablet with water and after that I take 2-3 teaspoon of yoghurt.

(INCTB02) A 14-year-old girl from India

**Table 3.** Frequencies and percentage of items ever mixed with 400- and 100-mg moxifloxacin to facilitate administration or improve taste.

	Mixed to facilitate administration		Mixed to improve taste	
	400-mg moxifloxacin (n = 29)	100-mg moxifloxacin (n = 36)	400-mg moxifloxacin (n = 29)	100-mg moxifloxacin (n = 36)
Nothing (%)	1 (3.4)	1 (2.8)	1 (3.4)	10 (27.8)
Water (%)	27 (93.1)	35 (97.2)	27 (93.1)	23 (63.9)
Yoghurt (%)	0	0	0	1 (2.8)
Porridge or pap (%)	0	0	0	0
Milk (%)	0	0	0	0 (0.0)
Tea or coffee (%)	0	0	0	1 (2.8)
Juice (%)	0	0	0	1 (2.8)
Banana (%)	0	0	0	1 (2.8)
Honey (%)	0	2 (5.6)	0	4 (11.1)
Sugar (%)	3 (10.3)	2 (5.6)	3 (10.3)	3 (8.3)
Other (%)	2 (6.9)**^A^**	2 (5.6)**^B^**	2 (6.9)**^A^**	2 (5.6)**^C^**

A Peanut butter, chocolate éclair/chocolate.

B Buko shake, anything that tastes sweet.

C Jaggery, buko shake.

Table 4 displays the comparison of the palatability of the 400- and 100-mg moxifloxacin at weeks 0 and week 2. About 31% disagreed/strongly disagreed with the statement ‘I/my child like/likes the taste of moxifloxacin’ for 100-mg moxifloxacin, and 48.3% reported same for 400-mg moxifloxacin – that is, the 100-mg moxifloxacin tasted better than the 400-mg formulation – though this difference was not statistically significant, *P* = 0.096. Table 5 displays the palatability; taste, smell, size, and dose volume of 100-mg moxifloxacin at weeks 2, 8, and 24. On almost all indicators, the proportion of participants reporting poorer acceptability is higher at weeks 8 and 24 than at week 2. For example, at week 2, 63.9% of the participants say that they strongly agree, agree, or are neutral about the statement ‘I/my child like/likes the taste of moxifloxacin’, but at week 24 only 20% of the participants respond in the same way. This trend is consistent across five of the seven items (noting that the item on smell is negatively worded, and therefore an increased proportion of strongly agree, agree, or neutral is negative), though it was not evaluated for statistical significance given the small sample size. On the remaining two items, almost all participants reported that they believed ‘(My child gets/I get) the whole dose of the moxifloxacin every time it is administered’ and ‘I believe the moxifloxacin is improving (my child’s/my) health’ at all three time points. The qualitative data suggest that when a participant compares the taste of 100-mg moxifloxacin to that of the 400-mg moxifloxacin (from which they had recently transitioned), then the 100-mg moxifloxacin compares favourably. However, once the memory of the 400-mg moxifloxacin formulations’ taste fades, and the participants think about the absolute taste of the 100-mg moxifloxacin (compared to ‘everyday’ tastes of food, etc.), then the taste is unpleasant. However, participants found ways to be resilient and cope, but even the 100-mg moxifloxacin remained unpalatable.

**Table 4.** Comparison of the palatability of 400- and 100-mg moxifloxacin at weeks 0 and 2 (n = 29).

	400-mg moxifloxacin strongly disagree or disagree (%)	100-mg moxifloxacin strongly disagree or disagree (%)	% difference (95% CI)	*P*-value
My child likes the taste of moxifloxacin.	14 (48.3)	9 (31.0)	−17.2 (−40.0, 5.5)	0.096
My child thinks the smell of moxifloxacin is gross/yucky.	12 (41.4)	18 (62.1)	20.7 (−8.7, 50.1)	0.134
The size of moxifloxacin makes it easy for my child to swallow.	7 (24.1)	2 (6.9)	−17.2 (−37.4, 3.0)	0.059
My child gets the whole dose of the moxifloxacin every time it is administered.	0	0	0.0 (−3.4, 3.4)	1.000
My child is upset every time moxifloxacin is administered.	18 (62.1)	20 (69.0)	6.9 (−9.8, 23.6)	0.317
I believe the moxifloxacin is improving (my child’s/my) health.	0	0	0.0 (−3.4, 3.4)	1.000
The side effects that (my child has experienced/I experienced) are unbearable.	23 (79.3)	28 (96.6)	17.2 (−3.0, 37.4)	0.059

**Table 5.** Palatability of 100-mg moxifloxacin at weeks 2, 8, and 24.

	100-mg moxifloxacin at week 2 (n = 36)	100-mg moxifloxacin at week 8 (n = 34)	100-mg moxifloxacin at week 24 (n = 30)
(My child likes/I like) the taste of moxifloxacin.			
Strongly agree, agree, or neutral (%)	23 (63.9)	15 (44.1)	6 (20.0)
Disagree or strongly disagree (%)	13 (36.1)	19 (55.9)	24 (80.0)
(My child thinks/I think) the smell of moxifloxacin is gross/yucky.			
Strongly agree, agree, or neutral (%)	16 (44.4)	15 (44.1)	16 (53.3)
Disagree or strongly disagree (%)	20 (55.6)	19 (55.9)	14 (46.7)
The size of moxifloxacin makes it easy for my child/me to swallow.			
Strongly agree, agree, or neutral (%)	32 (88.9)	30 (88.2)	22 (73.3)
Disagree or strongly disagree (%)	4 (11.1)	4 (11.8)	8 (26.7)
(My child gets/ I get) the whole dose of the moxifloxacin every time it is administered.			
Strongly agree, agree, or neutral (%)	36 (100.0)	32 (94.1)	30 (100.0)
Disagree or strongly disagree (%)	0	2 (5.9)	0
(My child is/ I am) upset every time moxifloxacin is administered.			
Strongly agree, agree, or neutral (%)	12 (33.3)	16 (47.1)	16 (53.3)
Disagree or strongly disagree (%)	24 (66.7)	18 (52.9)	14 (46.7)
I believe the moxifloxacin is improving (my child’s/my) health.			
Strongly agree, agree, or neutral (%)	36 (100.0)	33 (97.1)	30 (100.0)
Disagree or strongly disagree (%)	0	1 (2.9)	0
The side effects that (my child has experienced/I experienced) are unbearable.			
Strongly agree, agree, or neutral (%)	2 (5.6)	3 (8.8)	4 (13.3)
Disagree or strongly disagree (%)	34 (94.4)	31 (91.2)	26 (86.7)

#### Smell

For caregivers administering 100-mg moxifloxacin, 62% (18/29) reported that they ‘disagree/strongly disagree’ that the smell of the formulation is gross/yucky, as compared with 41% (12/29) who reported same for 400-mg moxifloxacin, indicating better acceptability of 100-mg moxifloxacin. A mother reported negative reaction from her child to the moxifloxacin, as her child found the smell too strong.

It gives me the feeling of ‘umay’ [nauseating feeling] … based on my experience… when I just smell the medication. Oh wow! ... Just the smell… I get the jitters… especially with moxifloxacin. The moment he [child] holds the cup, he can smell it – he starts to make a face.

(Mother of PHCTC01)

#### Size

For the 100-mg moxifloxacin, 89% (32/36) (Table 5) responded favourably to the statement ‘size of 100-mg moxifloxacin makes it easy for my children to swallow’. However, 24.1% of the participants reported disagreement that the size of the moxifloxacin made it easy for the child to swallow 400-mg moxifloxacin as compared with 6.9% for 100-mg moxifloxacin. This difference in disagreement was 17.2% (95% confidence interval: −37.4, 3.0, *P*-value = 0.059) (Table 4)

The new tablets [100-mg] are small and quite easy as there is no need to powdering them …. they just ‘dissolves’ into the water and administration is not ‘painful and tedious process’ like the old formulation [400-mg].

(INCTC04) Father of 8-month-old participant from India

#### General challenges with 100-mg moxifloxacin

Although 100-mg formulation of moxifloxacin is reported to be acceptable due to its ease of administration, due to dispersible nature and smaller size of tablets, caregivers faced fewer challenges suggesting that acceptability could be improved. The data from Table 4 reported that 31% of caregivers shared that their child was upset when they saw 100-mg moxifloxacin was to be administered as compared with 37.9 % of those who reported same response for 400-mg moxifloxacin. Qualitative data also reported similar emotional status of the children when they see and find that it is time to take medicine, as a caregiver from SA mentioned.

It was very difficult to give the medication to the child, and she was crying and screaming, refusing to swallow the dose administered with the syringe.

(SACTA03)

Few older children/caregivers mentioned increased pill burden of 100-mg as compared with the 400-mg moxifloxacin. They reported that as standard treatment, they were provided a single 400-mg tablet, but older children (higher weight band) now needed to take three to four tablets of the 100-mg moxifloxacin. The mother of an 8-year-old girl child stated that she has no problem administering the 100-mg moxifloxacin,

but it’s just the number of the daily doses has been increased (3 tablets) which is sometimes ‘intimidating’ for both of us

(INCTB01)

Despite challenges all caregiver participants perceived that both the 400-mg and the 100-mg formulation of moxifloxacin would be able to improve the child’s health.

## DISCUSSION

We found that the 100-mg moxifloxacin is more acceptable than the 400-mg moxifloxacin because of relative ease of preparation, but not due to improved palatability. This was true for both young children and their caregivers and for older children who swallowed the doses whole. The taste of moxifloxacin (regardless of the formulation) and smell when dispersed remain significant barriers to acceptability, across all settings and ages. A large proportion of children didn’t like the taste of the 100-mg moxifloxacin, which is consistent with clinical experience and other studies.^20^ There are still advantages to the 100-mg tablet due to its dispersible nature, including ease of preparation. Caregivers of young children reported having challenges in crushing the 400-mg moxifloxacin and end up administering a partial dose due to the reluctance of young children to swallow the medicine fully due to the bitter taste and strong smell. This finding is consistent with a study conducted at Cape Town, South Africa that reported poor acceptability of 400-mg moxifloxacin among children.^13^ Similar challenges of taste and duration of administration are reported as critical factors influencing acceptability of paediatric formulation in other studies on acceptability.^21,22^ The 100-mg formulation is a substantial improvement on this.

Similar to other acceptability studies^23,24^ caregivers and children in our study also adopted various strategies to overcome the challenges posed by poor palatability through bribery, physical force, administering the 100-mg moxifloxacin in solid form rather than dissolving it, and providing juice, sugar, or yoghurt to mask the after-taste of the medicine. Strategies were age-adjusted and used flexibly by parents. The WHO, in its second paediatric drug optimisation for TB (PADO-TB) meeting in 2023 retained moxifloxacin on the priority list, recognising that the palatability of currently available formulations remains sub-optimal.^25^ Moxifloxacin palatability for children should remain a high priority for drug manufacturing companies to improve acceptability.

One of the key strengths of our study is that it used longitudinal qualitative and quantitative data at three sites in three countries with diverse cultural and geographical settings providing contextual variability. Limitations include the small sample size (especially for the quantitative findings) that limits generalisability.

## CONCLUSION

This study provides evidence of improved acceptability of the 100-mg dispersible moxifloxacin in diverse high-TB-burden settings. Ease of administration and smaller pill size emerged as significant factors for improved acceptability.

## Supplementary Material


