# Supplementary material for: Acceptability of 100-mg moxifloxacin in children with rifampicin-resistant TB in three high-burden countries
Source: IJTLD Open. 2025 Oct 10;2(10):597–603. doi: 10.5588/ijtldopen.25.0365 (PMC12517267; doi:10.5588/ijtldopen.25.0365)
Supplement: Supplementary file 1 [file ijtldopen25-0365_supplementarydata1.pdf]

Supplementary material 1. Acceptability questionnaire

	Past experiences with routine 400 mg moxifloxacin ingestion/administration [WEEK 0]		
S3_Inst	I am now going to ask you a few questions about you/your child's general experiences with the standard moxifloxacin formulation. These tablets are usually given to children as part of their TB Treatment (show picture of routine formulation of moxifloxacin).		
S3_Taken	Have you/your child ever taken those tablets (the routine formulation of moxifloxacin tablets)?	1, Yes<br>2, No<br>3, I don't know	
S3_Admin	How are the [routine 400mg] formulation tablets usually administer to the child/you?	1, Swallowed whole<br>2, Chewed	3, Grind finely and mix with liquid<br>4, Grind finely and mix with food
S3_MixSwall	Which of the following are usually mixed with the tablet *to make it easier to swallow/take all of the dose*? Please indicate all that apply.	1, Nothing<br>2, Water<br>3, Yoghurt<br>4, Porridge/pap<br>5, Milk<br>6, Tea/Coffee	7, Juice<br>8, Banana<br>9,Honey<br>10, Sugar<br>99, Other (specify)
S3_MixTaste	Which of the following are usually mixed with the tablet *to hide the taste of the medicine or make it taste better for the child?* Please indicate all that apply.	1, Nothing<br>2, Water<br>3, Yoghurt<br>4, Porridge/pap<br>5, Milk<br>6, Tea/Coffee	7, Juice<br>8, Banana<br>9, Honey<br>10, Sugar<br>99, Other (specify)
S3_Inst2	I am now going to read you a series of statements about the routine moxifloxacin formulation tablet. For each statement I want you to answer either: "Strongly agree", "Agree", "Neutral", "Disagree", or "Strongly disagree". Remember I am asking about the [routine]er moxifloxacin formulation, not the new one from the study. Show participant image of scale labelled [Strongly agree - Strongly disagree] at the beginning of the section and again as needed.		
S3_Taste	I/My child really *likes the taste* of the [routine] formulation moxifloxacin tablets	1, Strongly agree<br>2, Agree<br>3, Neutral	4, Disagree<br>5, Strongly disagree
S3_Smell	I/My child thinks that the *smell* of the [routine] formulation of moxifloxacin is *gross/yucky*.	1, Strongly agree<br>2, Agree<br>3, Neutral	4, Disagree<br>5, Strongly disagree
S3_Size	The *size* of the [routine] formulation of moxifloxacin tablets makes it *easy* for me/my child *to swallow*.	1, Strongly agree<br>2, Agree<br>3, Neutral	4, Disagree<br>5, Strongly disagree
S3_WholeDos	I am sure that I/my child gets the *whole dose* (as prescribed by the doctor) of the	1, Strongly agree	4, Disagree

	[routine] formulation of moxifloxacin tablets every time that it is administered.	2, Agree<br>3, Neutral	5, Strongly disagree
S3_Tension	*Administering* the [routine] formulation of moxifloxacin tablets has become a source of *unhappiness or tension* in my family.	1, Strongly agree<br>2, Agree<br>3, Neutral	4, Disagree<br>5, Strongly disagree
S3_Upset	I/My child is *upset* every time the [routine] formulation of moxifloxacin tablets are administered	1, Strongly agree<br>2, Agree<br>3, Neutral	4, Disagree<br>5, Strongly disagree
S3_ImprHea	I believe that the [routine] formulation of moxifloxacin tablets are *improving my/my child's health*.	1, Strongly agree<br>2, Agree<br>3, Neutral	4, Disagree<br>5, Strongly disagree
S3_SideEff	The *side effects* that my child has experienced from the [routine] formulation of moxifloxacin are *unbearable*.	1, Strongly agree<br>2, Agree<br>3, Neutral	4, Disagree<br>5, Strongly disagree
S4_ING	Experiences with novel 100 mg dispersible moxifloxacin formulation ingestion/administration **[WEEK 2, 8, 24]**		
S4_Inst	I am now going to ask you a few questions about you and your child's general experiences with the novel moxifloxacin 100mg formulation. These are the small white tablets with the M and the '100' on it (Show image)		
S4_Admin	How are the [novel moxifloxacin 100mg formulation] tablets usually administered to you/the child?	1, Swallowed whole<br>2, Chewed	3, Dispersed in water<br>4, Other(specify)
S4_MixSwall	Which of the following are usually mixed with the [novel moxifloxacin 100mg formulation] tablets to make it easier to swallow/take all of the dose? Please indicate all that apply.	1, Nothing<br>2, Water<br>3, Yoghurt<br>4, Porridge/pap	5, Milk<br>6, Tea/Coffee<br>7, Juice<br>99, Other (specify)
S4_MixTaste	Which of the following are usually mixed with [novel moxifloxacin 100mg formulation] tablets to hide the taste of the medicine or make it taste better for the child? Please indicate all that apply.	1, Nothing<br>2, Water<br>3, Yoghurt<br>4, Porridge/pap	5, Milk<br>6, Tea/Coffee<br>7, Juice<br>99, Other (specify)
S4_Inst2	I am now going to read you a series of statements about the novel moxifloxacin formulation (small white tablets with the M and the '100' on it). For each statement I want you to answer either: "Strongly agree", "Agree", "Neutral", "Disagree", or "Strongly disagree".		
S4_Taste	I/My child really likes the taste of the [novel moxifloxacin 100mg formulation] tablets.	1, Strongly agree<br>2, Agree<br>3, Neutral	4, Disagree<br>5, Strongly disagree
S4_Smell	I/My child thinks that the smell of the [novel moxifloxacin 100mg formulation] tablets, is gross/yucky.	1, Strongly agree<br>2, Agree<br>3, Neutral	4, Disagree<br>5, Strongly disagree

S4_Size	The size of [novel moxifloxacin 100mg formulation] tablets makes it easy for me/my child to swallow.	1, Strongly agree<br>2, Agree<br>3, Neutral	4, Disagree<br>5, Strongly disagree
S4_WholeDos	I am sure that I/my child gets the whole dose (as prescribed by the doctor) of the [novel moxifloxacin 100mg formulation] tablets every time that it is administered.	1, Strongly agree<br>2, Agree<br>3, Neutral	4, Disagree<br>5, Strongly disagree
S4_Tension	Administering the [novel moxifloxacin 100mg formulation] tablets has become a source of unhappiness or tension in my family.	1, Strongly agree<br>2, Agree<br>3, Neutral	4, Disagree<br>5, Strongly disagree
S4_Upset	I/My child is upset every time the [novel moxifloxacin 100mg formulation] tablets is administered	1, Strongly agree<br>2, Agree<br>3, Neutral	4, Disagree<br>5, Strongly disagree
S4_ImprHea	I believe that [novel moxifloxacin 100mg formulation] tablets are improving my/my child's health.	1, Strongly agree<br>2, Agree<br>3, Neutral	4, Disagree<br>5, Strongly disagree
S4_SideEff	The side effects that I/my child has experienced from the [novel moxifloxacin 100mg formulation] tablets are unbearable.	1, Strongly agree<br>2, Agree<br>3, Neutral	4, Disagree<br>5, Strongly disagree

Supplementary material 2: Discussion Guide: CATALYST
DO NOT CITE WITHOUT PERMISSION

Discussion guide

Clofazimine and moxifloxacin PK, safety, and AccepTAbiLitY for paediatric TB treatment. (CATALYST)

Purpose: This document is to be completed by the research staff completing discussions with key informants who form part of the CATALYST study. The aim of the study is to understand children receiving Rifampicin resistant TB (RR-TB) treatment and their caregivers' perceptions of treatment palatability and acceptability and to understand how their experiences develop over the course of the treatment journeys. The discussions should be completed with either the caregiver or the child participant, depending on the age of the participant and their ability to participate.
Note: The activities and questions in this discussion guide are expected to be completed in 3-4 interviews. The first of the interviews will likely coincide with their enrolment visit. The remaining discussions/topic areas will be conducted at approximately 4, 16, and 24 weeks after enrolment.
Expected time needed per use: 45~60 minutes per interview.

Visit 1: Week 0 (facility)

Kinship map activity

Aim: To draw a picture that represents (1) the participants' "family" (biological and non-biological), (2) the types of relationships between each member of that "family", and (3) cohabitation/co-residence of people ("family" and non-family) with the participant.

1. Researcher to tell the participant: "*We are going to be doing a Kinship Map with you today. A kinship map is like a family tree, or something we use to draw your family and household. It will help us to see who you live with and help us to get to know you and your family. We are going to draw the family tree or kinship map together*." If the child is old enough, they can participate with the help of the caregiver/adult.
2. Ask the participant to create a list of people that are part of their family and household.
3. Ask about co-residence/co-habitation.

   Additional probes:

1. Please indicate, which members of the family are aware of the child's diagnosis?
2. Please indicate which family members have ever been affected by TB or RR-TB?

Household floorplan activity

Aim: To 1) draw the infrastructure of the participant's house and spaces that they use on a daily basis; 2) understand the participant's perception of their home and how they relate to their house/home; 3) understand the participant's perception of how other people experience their house/living space and, overall, how it relates to illness management. The participant should either be the child if they are able (with assistance from the caregiver where needed) or the adult caregiver, as appropriate.

1) Researcher to participant: "*As part of this activity we will be drawing a floorplan for your house to better understand the layout of your home and how different people use the space. This will help us understand if the ways in which households influences the ways in which health or illnesses are managed.*"
2) Draw an outline of their house and the yard overall; draw the inner lines of the house; draw the furniture; explain how different spaces are used/shared

Visit 2: Week 4 (facility)

Illness narrative and timeline activity

Aim: To understand the illness journey of the participant from the beginning (pre-diagnosis through diagnosis and treatment) and how the journey evolved overtime.

1. "*We are going to be doing an illness journey timeline activity. An illness journey allows researchers and health workers to understand your (or your child's) unique experiences of living with TB. This also allows researchers and health workers to understand the role TB plays in your life and the impact that TB has had on it. The timeline will allow us to see the 'main/important events' that has occurred in your/your child's life*."
2. Ask the participant to tell you the story of how they (child patient) managed to become enrolled in this study. Plot the points such as diagnosis, symptoms, referrals, and major events next to their age.
3. Ask the participant to walk you through their journey of when and at what age they had their first experience/encounter with TB and ask them to note this on the page/timeline.

Illness narrative and timeline follow-up questions: Treatment (Repeated at Visit 3 and 4)

Aim: Building on the illness narrative/timeline, to explore the ways in medications are viewed and experienced by child patients (or their caregivers). This includes exploring 1) the ways in which the medications are administered; 2) overall perceptions of the medications; 3) overall acceptability/palatability of the medications.

1. Explain to participant: "*We have discussed your treatment and illness journey up to now. We can now talk about the new treatment that you are currently taking.*"
2. Please describe all the medication you/child are taking as part of the study treatment and how they are taken (packaging, taste, feel, etc.)
3. What have been the difficult things about taking/giving these treatments?

Body map activity

Aim: To draw a picture that represents (1) the participants' body ; (2) to explore how the participant perceives their body; 3) to understand how the participants experience their health condition(s); and 4) to explore the participant's emotional-physical-social experience as a result of their health condition(s), including the treatment they receive.

1) "*We would like to understand how RR-TB has affected your body. To do that, we are going to draw a 'body map'. A body map is a life-sized (or printed) outline of your body where you can fill in or draw details*."
2) Ask the participant to mark all the places on their body where TB affects them/their child.
3) Ask the participant to expand on how the treatment makes their body feel.

<u>Visit 3: Week 16 (facility)</u>

Illness narrative follow-up questions: Treatment (Repeat activity – as per visit 2)

Health beliefs Action activity

Aim: The aim of this activity is 1) to understand the thought process that influences health seeking behaviours in participants; 2) to understand how perceptions of susceptibility and severity of disease influence choices to prevent disease and adhere to treatment; and 3) to understand how individual's perceptions of how they SHOULD behave, influences their eventual decisions to either prevent a

disease and/or adhering to treatment. Either the child or the caregiver should complete the activity, depending on the age/capabilities of the child.

1) Tell the participant" *"As part of this next activity, we want to find out what are the things that influence how people think about getting health care, how people understand illnesses, including how bad illness can be or how likely they are to become ill, and what people think about what people are and should be doing when taking treatment."*
2) Areas to explore:
    a. Susceptibility
    b. Severity
    c. Control over outcomes (general)
    d. Ability to implement behaviour (specific)
    e. Descriptive normative expectations
    f. Injunctive normative expectations

Health beliefs

Aim: To understand how participant makes sense of and manage their health. By discussing the different health systems that participants engage with, we are better able to understand how participants make decisions around accessing care or treatment. To do this we will ask participants to (1) identify the different health systems they access and (2) explore how participants engage with these health systems for varying health conditions.

Visit 4: Week 24 (Household visit)

Illness narrative follow-up questions (Repeat activity – as per visit 2 and 3)

Household income/expenses activity

Aim: Exploring how income is generated and to explore the effects of TB on household socio-economic status. People living in under-resourced communities experience a greater burden of disease. Through this activity, we explore and illustrate the linkages between socio-economic circumstances and how households manage paediatric TB. This activity should be conducted with the caregiver of the child patient.

1) "*We are going to do a household income/expenses activity. A household income activity includes describing and showing us how money or other resources are brought into and spent from your household. The reason why we want to know this is to help us understand the ways in which RR-TB affects the finances of a household. Your story will help to develop strategies and programmes to better help persons with RR-TB. One way of showing how money or resources is coming into your household is by pretending that all the incomes/expenses are part of a pie/cake. We want to understand what the different 'slices' (incomes) are that form part of the pie/cake.*"
    a. List of all the household income sources and expenses
    b. Effect of the child's TB diagnosis.
    c. How/if their household has been affected by the COVID-19 pandemic/ lockdown measures.

Parameters of ambitions activity

AIM: To understand the parameters of ambition of participants which take the form of hopes, dreams, ambitions, and fears. By discussing these parameters, we may illicit the motivations for general health seeking behaviour such as pursuing/not pursuing health care, adherence, and treatment acceptability.

1) "*We have done several timeline activities about things that have happened in the past. Now we would like to do a timeline about the future*". Ask the participant to draw a timeline (using the standard timeline template) to indicate their plans and dreams for the future of their child (if caregiver) and child (if old enough to participate).

Thank you so much for talking to us. Is there anything else we need to know?
